# Stereoisomer-Independent Stable Blue Emission in Axial Chiral Difluorenol

**DOI:** 10.3389/fchem.2021.717892

**Published:** 2021-09-03

**Authors:** Mengna Yu, Xiong Jia, Dongqing Lin, Xue Du, Dong Jin, Ying Wei, Linghai Xie, Wei Huang

**Affiliations:** ^1^ Center for Molecular Systems and Organic Devices (CMSOD), State Key Laboratory for Organic Electronics and Information Displays, Institute of Advanced Materials (IAM), Nanjing University of Posts and Telecommunications, Nanjing, China; ^2^ Frontiers Science Center for Flexible Electronics (FSCFE), MIIT Key Laboratory of Flexible Electronics (KLoFE), Northwestern Polytechnical University (NPU), Xi’an, China

**Keywords:** axial chiral, deep blue, stereoisomer, difluorenol, spirobifluorene

## Abstract

Bulky conjugated molecules with high stability are the prerequisite for the overall improvement of performance in wide-bandgap semiconductors. Herein, a chiral difluorenol, 2,2′-(9,9′-spirobi[fluorene]-2,2′-diyl)bis(9-(4-(octyloxy)phenyl)-9H-fluoren-9-ol) (DOHSBF), is set as a desirable model to reveal the stereoisomeric effects of wide-bandgap molecules toward controlling photophysical behavior and improving thermal and optical stability. Three diastereomers are obtained and elucidated by NMR spectra. Interestingly, the effect of modifying the stereo-centers is not observed on optical properties in solutions, pristine films, or post-treated film states. All three diastereomers as well as the mixture exhibit excellent spectral stability without undesirable green emission. Therefore, this stereoisomer-independent blue-emitting difluorenol will be a promising candidate for next-generation wide-bandgap semiconductors that would have extensive application in organic photonics.

## Introduction

Organic wide-bandgap blue-emitting semiconductors have attracted more attentions in industrial and fundamental research in information display and solid lighting (Friend et al., 1999; Xie et al., 2012). However, there is one key tough obstacle needed to be overcome before achieving comparable performance with the inorganic counterparts and that is stability (Honmou et al., 2014; Heeger, 2010; Spano and Silva, 2014). The poor color purity and low spectral stability are usually derived from aggregation-induced excimer emission (Farinola and Ragni, 2011; Knaapila and Monkman, 2013), ketone formation (Bliznyuk et al., 1999; Sims et al., 2004), distorted conformation, or entanglement chains (Liu et al., 2016). In addition to device performance, improving the stability of blue luminescent molecules has been a long-standing challenge for plastic electronics. Molecular bulks are favorable for the thermal and morphological stability in organic wide-bandgap semiconductors with potential applications in both information and energy electronics. Bulky groups are the sp^3^ carbon-containing groups which possess the steric hindrance effect, and functionalized bulky groups were introduced into optoelectronic materials which acted as the suppression of intermolecular force, resulting in the morphological stability (Li et al., 2018; Li et al., 2016; Yu et al., 2019). Therefore, designing bulky conjugated molecules is the prime requirement for light-emitting applications. In the past, the spirobifluorene structure was introduced as the bulk unit to effectively enhance structural rigidity, provide better photothermal stability, and avoid fluorescence quenching (Nakagawa et al., 2012; Li et al., 2016). Fluorene-based derivatives were positioned as an important class of blue-emitting semiconductors for their pure blue emission, high photoluminescence quantum efficiency (PLQE), and easy modification (Xie et al., 2012; Yu et al., 2019; Zhu et al., 2019). As a functional position of fluorene units, the chiral sp3 carbon at the ninth position of the fluorene monomer is an active site for preparing variable stereoisomers and tuning the optoelectronic structure (Karimov and Hartwig, 2018; Pitre et al., 2019). Previously, we demonstrated a supramolecular chiral oligofluorenol, 2,2′-(spiro[fluorene-9,9′-xanthene]-2,7-diyl)bis(9-(4-(octyloxy)phenyl)-9H-fluoren-9-ol) (2O8-DPFOH-SFX), to investigate the stereoisomerism–property relationship of conjugated aromatic molecules and explore optoelectronic properties (Yuan et al., 2018; Yu et al., 2019). Therefore, precisely controlling the stereochemistry of organic molecules is an important and effective approach for achieving unique photophysical properties.

As chiral structures play a crucial role in sustaining lives, asymmetric synthesis of chiral structures has attracted considerable attention from organic synthetic chemists in many research groups. Early studies mainly focused on central chirality (central atoms with different substituents). Different from molecules with central chirality (point chirality), axial chirality resulting from the steric hindrance of the rotation of the bonds (Nguyen, 2018), has attracted tremendous attention and intensive efforts. The history of the axial chiral structure could date back to 1920s, when scientists discovered special isomerism of the biphenyl structure (Christie and Kenner, 1922). By 1969, Prelog et al. prepared the first enantiomeric pure spirobifluorene and inspired plenty of scientists to investigate chiral axial compounds, which become a shaft in the research of chiral spiro compounds (Haas and Prelog, 1969). To date, axial chiral molecules not only have been widely used in organic reactions, such as kinetic resolution, asymmetric catalysis, cyclamation/addition, direct aromatization, and chiral recognition, but also exhibit promising application in optoelectronic fields like molecular electronic devices, semiconductors, light-emitting devices, and solar cells (Han et al., 2017; Mishra et al., 2017; Wang and Tan, 2018; Liu Z. S. et al., 2020). Inspired by the supramolecular steric hindrance (SSH) effect (Li et al., 2018), herein, we try to propose a novel strategy based on the molecular integration of steric bulk groups and axial chiral synthons into one functional molecule. We present difluorenol (DOHSBF), which consist of an axial chiral spirobifluorene and two chiral sp3 carbon atoms bearing a phenyl ring and a hydroxyl moiety. Unexpectedly, DOHSBF shows three different stereoisomers, which display stereoisomer-independent stable blue emission.

## Result and Discussion

### Material Synthesis and Characterization

**FIGURE 1:**
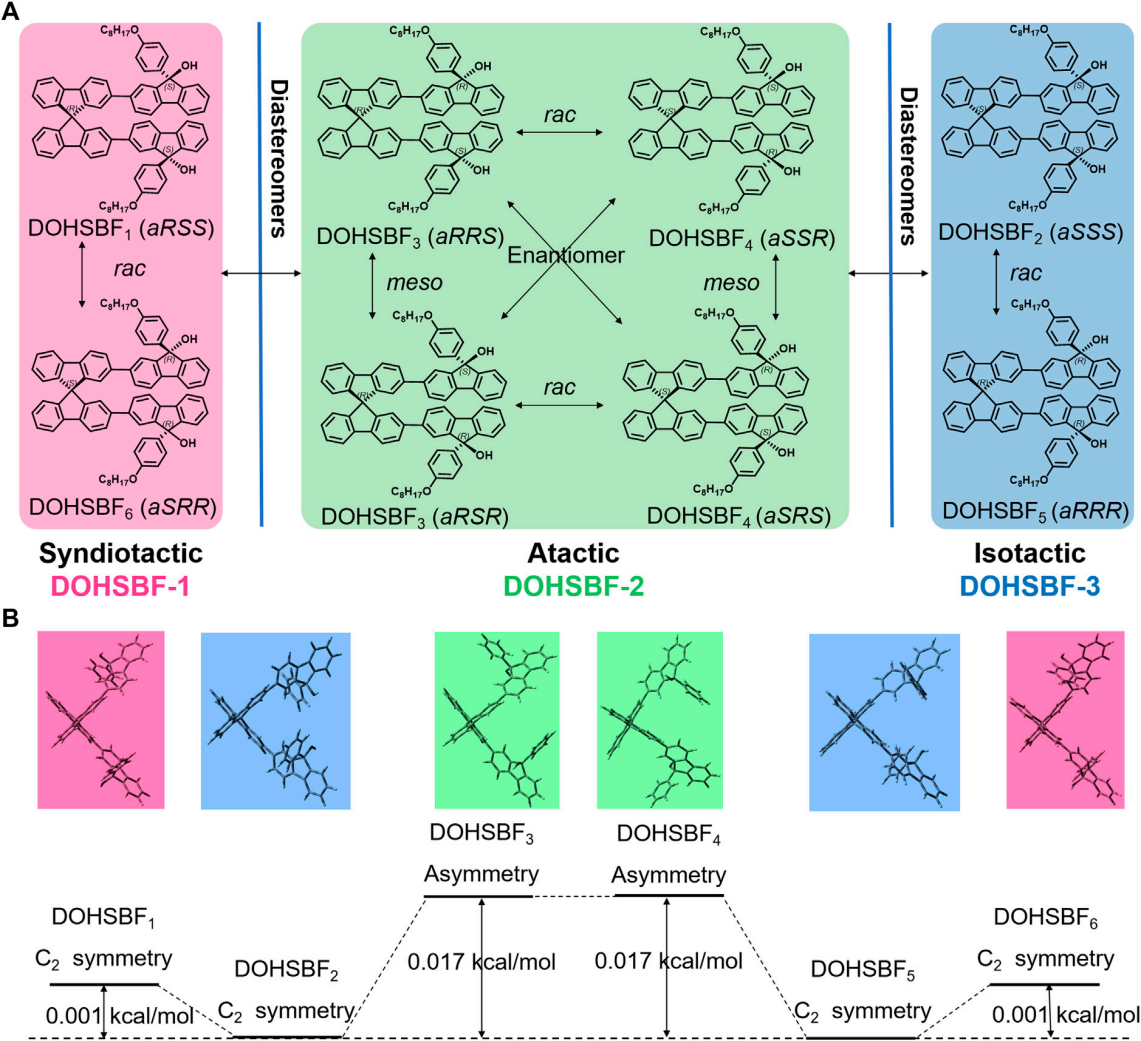
**(A)** Theoretical configurations of DOHSBF. **(B)** Energy calculation comparison of possible theoretical stereoisomers of DOHSBF (alkyl chains are omitted) by Gaussian 09 at the B3LYP/6-31G(d) level.

The DOHSBF unit (without alkyl chains) consists of two tertiary alcohols and one spirobifluorene, which form the composition of C_79_H_72_O_4_. Theoretically, DOHSBF has six stable stereoisomers due to the three chiral sp3 carbon atoms and the orientation of benzene rings in the molecule, which are DOHSBF_1_ (*aRSS, C*_*2*_ symmetry), DOHSBF_2_ (*aSSS, C*_*2*_ symmetry), DOHSBF_3_ (*aRRS,* Asymmetry), DOHSBF_4_ (*aSRS,* Asymmetry), DOHSBF_5_ (*aRRR, C*_*2*_ symmetry), and DOHSBF_6_ (*aSRR, C*_*2*_ symmetry) (Figure 1A and Supplementary Figure S1), making it an excellent candidate to investigate the stereoisomeric effect of the fluorenol system. These six isomers can be distinguished by different chirality of spirobifluorene and the orientation of benzenes. They can be divided into two pairs of racemes and two mesomers. Among these six stable stereoisomers obtained from the structural optimization *via* quantum calculation, DOHSBF_1_, DOHSBF_2_, DOHSBF_5_, and DOHSBF_6_ seem to show centrosymmetric backbones (*C*_*2*_ symmetry), which is beneficial to define the actual structures among the isomers. In addition, the optimization results of DOHSBF (Figure 1B) show that the energies of DOHSBF_1_ (*aRSS*) and DOHSBF_6_ (*aSRR*) structures are the lowest among the six diastereomers. Taking the energy of the *aRSS* structure (0 kcal/moL) as a reference, the energy of the other four diastereomers is 0.40 kcal/moL (DOHSBF_2_, *aSSS*), 0.48 kcal/moL (DOHSBF_3_, *aRRS*), 0.44 kcal/moL (DOHSBF_4_, *aSRS*), and 0.48 kcal/moL (DOHSBF_5_, *aRRR*).

**FIGURE 2:**
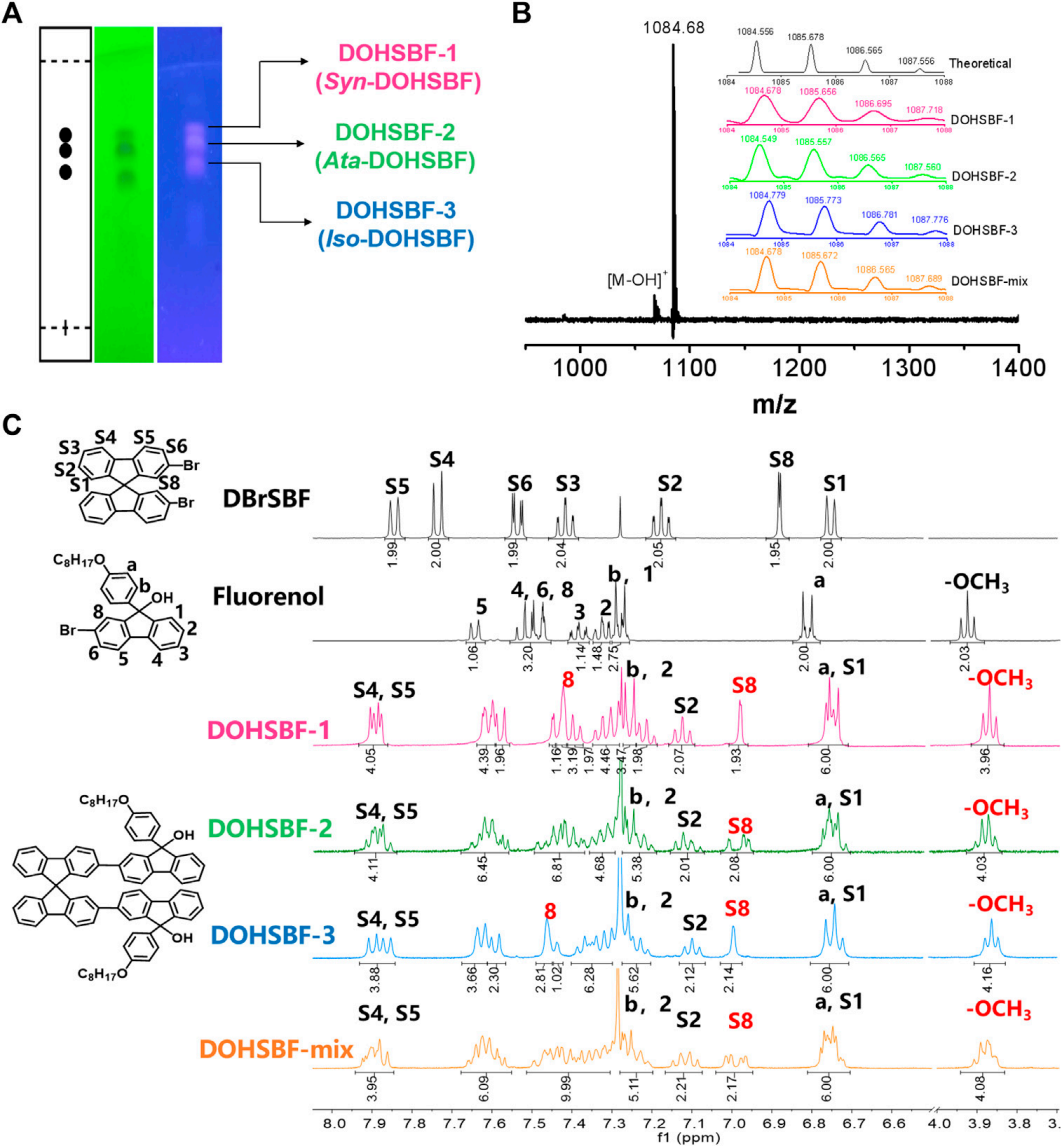
**(A)** TLC plate images of the DOHSBF reaction mixture under the 254 nm lamp (left) and the 365 nm lamp (right) (the mobile phase is PE: DCM (1:3) and the TLC plate has been placed into jar twice). **(B)** MALDI-ToF-MS spectra of DOHSBF. **(C)** ^1^H NMR of DBrSBF, fluorenol, and DOHSBF in the aromatic region.

Three pairs of DOHSBF diastereoisomers were synthesized *via* the Suzuki–Miyaura coupling reaction from 2,2′-spirobifluorene (with mixed a*S*- and a*R*-axis-chirality) and can be isolated *via* thin-layer chromatography (Figure 2A). The isomeric features are examined through matrix-assisted laser desorption/ionization time of flight mass spectroscopy (MALDI-ToF-MS). In Figure 2B and Supplementary Figure S2, the mixed DOHSBF samples only exhibit the molecular weight of 1,084.68 m/z, which is almost identical to isomers 1, 2, and 3 with the molecular weight of 1,084.68, 1,084.55, and 1,084.78, respectively. These results are consistent with the mass simulation of the molecular formula C_79_H_72_O_4_ (1,084.56). Furthermore, these isomers properties are reconfirmed by the same number of hydrogen atoms at the aromatic groups, according to the ^1^H NMR spectra (Figure 2C and Supplementary Figures S3–6) that exhibits the approximately equivalent integration at 7.95–7.85 ppm (4H, assigning to the S4 and the S5-position at SBF moiety), 7.70–7.55 ppm (6H), 7.50–7.20 ppm (16H), 7.15–7.05 ppm (2H, at the S2-position), 7.05–6.95 ppm (2H, at the S8-position), and 6.85–6.70 ppm (at the a-site on fluorenol and the S1-position on the SBF group). Even so, there are some subtle differences in chemical shift of hydrogen signals, probably derived from the integrated tactic effects of SBF-based axis chirality (Hamada et al., 2020) and asymmetric fluorenol chirality (Yuan et al., 2018; Yu et al., 2019; Wei et al., 2019). At 7.00 ppm, isomers 1 and 3 possess the singlet peak of S8 at 6.98 and 6.96 ppm, respectively, while isomer 2 exhibits the splitting multiple S8 peaks in this region. The splitting signals are also observed in 3.95–3.80 ppm (assigning to alkoxyl chains pendant on fluorenol groups) and 7.15–7.05 ppm for isomer 2, which suggests that the asymmetric backbones is consistent with the *meso*-DOHSBF type (DOHSBF_3_ and DOHSBF_4_, in Figure 1A). The symmetric structures without splitting in isomer 1 are assigned to *rac*-DOHSBF types, as is agreement with the simulated *C*_*2*_-symmetric results (Figure 1B). These results are consistent with SFX-based difluorenol building blocks where the *meso*-configuration exhibits the asymmetrically splitting feature on proton resonance (Yuan et al., 2018; Yu et al., 2019). Meanwhile, it is suggested that SBF-based axis chirality does not additionally break the symmetry of steric structures, as is in agreement with the tactic effects of other axis-chiral groups such as chiral binol-phosphate esters (Xiang et al., 2020) and 9,9′-spirobifluoren-derivates (Haas and Prelog, 1969).

### Thermal and Electrochemical Properties

**FIGURE 3:**
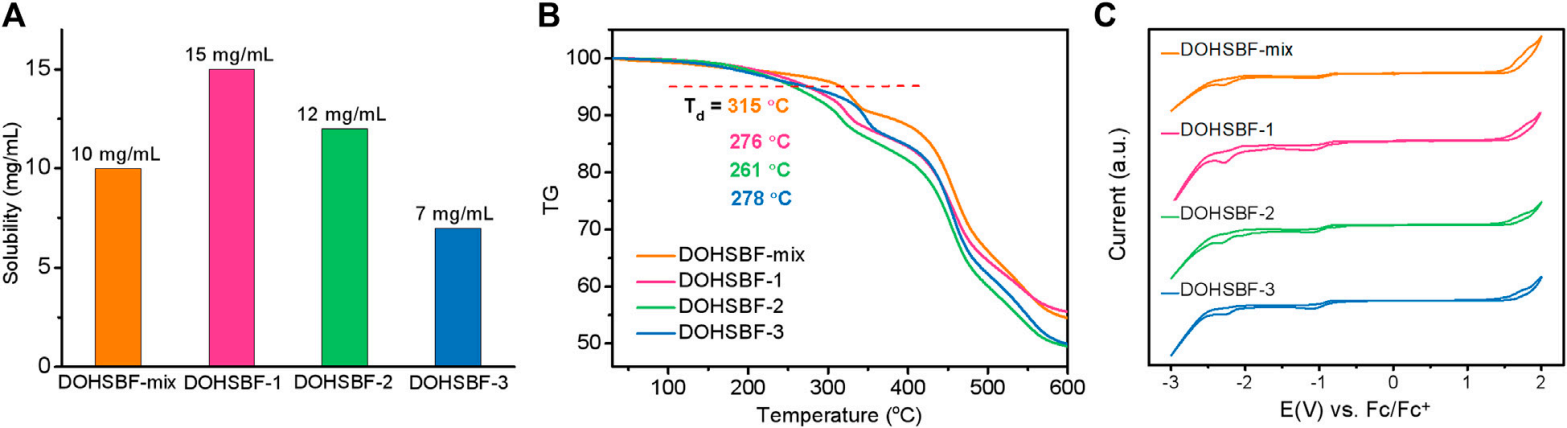
**(A)** The solubility of DOHSBF in dichloromethane. **(B)** TGA curves and **(C)** cyclic voltammogram of DOHSBF.

As depicted in Figure 3A and Supplementary Figure S7, all three stereoisomers show good solubility in organic solvents, such as dichloromethane, tetrahydrofuran, chloroform, and ethyl acetate, and exhibit a deep blue color. But the solubility of DOHSBF-1 is better than DOHSBF-2 in dichloromethane, and then DOHSBF-3. The X-ray powder diffraction (XRD) patterns in Supplementary Figure S8 show no apparent diffraction peaks, indicating that when pure, all the isomers formed amorphous films. The thermal stability is confirmed using the thermogravimetry analysis (TGA) and differential scanning calorimetry (DSC) measurements. Decomposition temperatures (5% weight loss, termed T_d_) recorded in order are mixture (315°C) > isomer-3 (278°C) > isomer-1 (276°C) > isomer-2 (261°C) (Figure 3B and Supplementary Figure S9). These high T_d_ values suggested good thermal stability of DOHSBF for its application in optoelectronic devices, and the mixture of difluorenol possesses the enhanced stability during the evaporation process. The DSC curves indicate that three pure difluorenol derivatives show no glass phase transition and melting point by heating to 178°C, but the mixture presents an inconspicuous glassy transition temperatures (T_g_) at 114°C (Supplementary Figure S10). The cyclic voltammetry (CV) measurement was carried out to investigate the electrochemical oxidation and reduction behaviors of DOHSBF and estimated the corresponding HOMO and LUMO energy levels. The CV curves of DOHSBF with different forms are showed in Figure 3C, with the electrochemical properties of DOHSBF listed in Supplementary Table S1. The oxidation onset potential is recorded at 1.49, 1.54, 1.50, and 1.48 V for DOHSBF-mix, DOHSBF-1, DOHSBF-1, and DOHSBF-3 vs. Ag/Ag^+^, respectively. As a result, according to the empirical formula E_HOMO_ = −(E_ox_−E_Fc_)−4.8 eV, the corresponding highest occupied molecular orbital (HOMO) energy levels of the four compounds were −6.26, −6.31, −6.27, and −6.25 eV, respectively, and the lowest unoccupied molecular orbital (LUMO) energy levels were measured to be −2.75, −2.70, −2.63, and −2.67 eV, respectively. As a result, the electrochemical band gaps were calculated to be 3.51, 3.61, 3.64, 3.58 eV, respectively. The energy levels revealed that configuration diversity induced by the chiral structure in fluorenol-based materials in the solution state seems to show slight effects on the electronic structure. The electrochemical results demonstrated that all the DOHSBF molecules show a wide-bandgap characteristic.

### Photophysical Properties of DOHSBF in Various States

**FIGURE 4:**
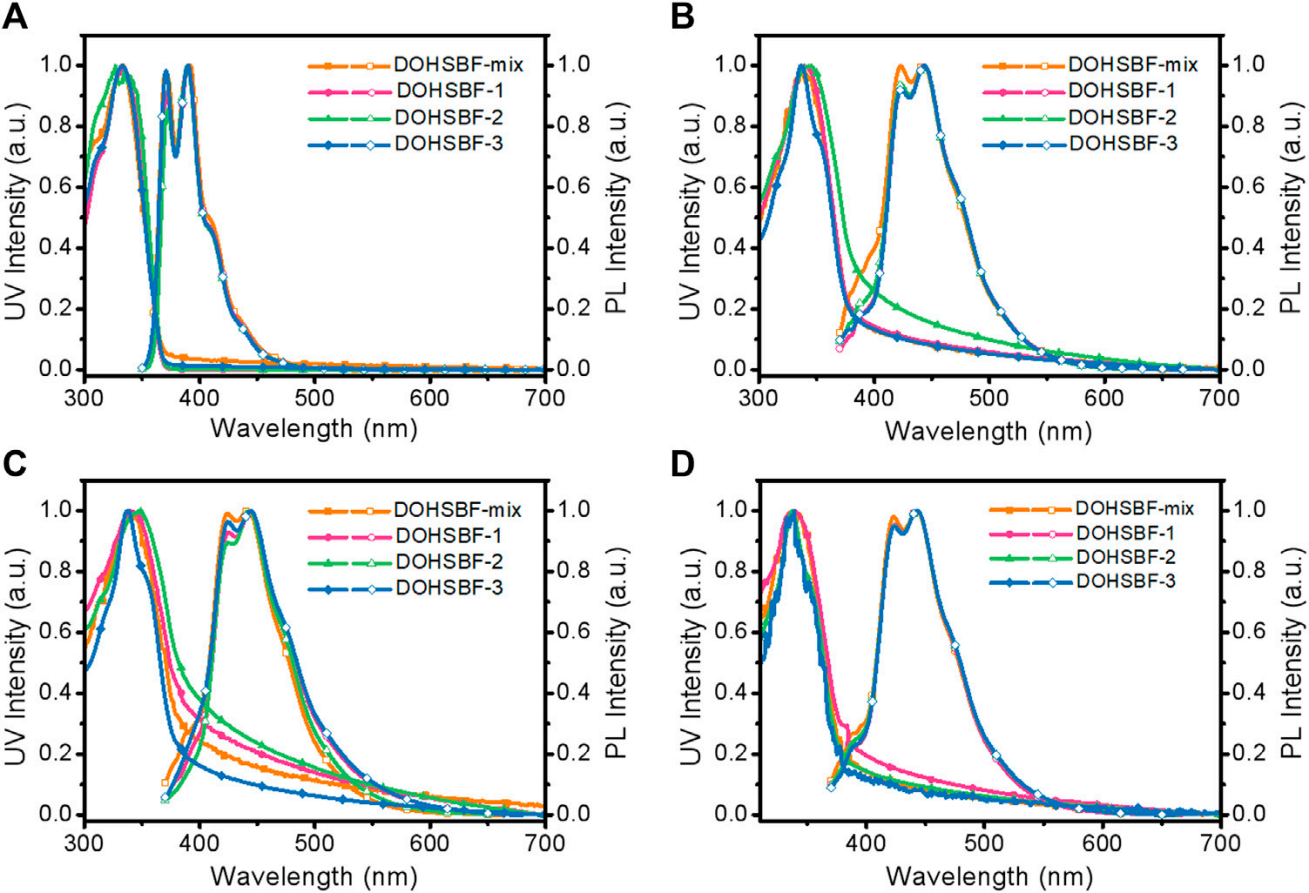
Optical properties of DOHSBF in various states. UV-vis absorption and photoluminescence (PL) spectra of DOHSBF **(A)** dilute solutions, **(B)** spin-coated pristine films, **(C)** annealed films at 220°C for 10 min, and **(D)** photooxidation films, respectively.

The absorption and emission spectra of DOHSBF in solutions, pristine films, and post-treated film states were investigated to further disclose the diastereomeric effects on optical properties (Figure 4; Table 1). Dilute solutions of tetrahydrofuran at a concentration of 10^−5^ mg/ml were used to investigate the photophysical properties of the four different chiral forms. As depicted in Figure 4, all the isomers show similar maximum absorption peaks at 333 nm in the solution state and 338 nm in the film state due to similar conjugated backbone structures. As depicted in Figure 4A, the PL spectrum of DOHSBF-mix in diluted solution was composed of three well-resolved emission peaks at around 371, 390, and 410 nm, attributable to the 0–0, 0–1, and 0–2 vibrational transitions of single molecule, respectively, having similar solution spectra in the other three isomers. The emission profiles of DOHSBF in spin-coated pristine films are shown in Figure 4B. Similarly, the emission spectra of them in the film state present three emission bands at about 423, 443, and 471 nm, and the corresponding 0–1 transition in the film are bathochromic to 443 nm, compared to the solution states. In addition, thermal annealing at high temperature in air was further conducted to study the spectral stability for the isomer films. It can be seen from the Figure 4C and the Supplementary Figure S11 that there is almost no change in the UV-vis spectra of DOHSBF films after annealing in air for 10 min. With regard to fluorescence emission spectra, there is no difference after annealing, illustrating that the DOHSBF has relatively good thermal stability. Compared to the conventional thermal stability, the oxidation stability of luminescent films has been rarely addressed in the last several decades, which is the most important factor determining practical optoelectronic application in the future. Herein, we make a further research study on the influence of chiral forms by photooxidation of pristine spin-coated films. Experimental procedure for photooxidation measurement involves exposing the pristine films under an ultraviolet lamp (365 nm) irradiation for 30 min. As presented in Figure 4D, no obvious change of absorption and emission behaviors was found in all four samples, and the green emission band with the I_green_/I_blue_ ratio (the ratio of emission intensities at 550 and 443 nm) is very low, indicating its excellent deep blue emission spectral stability without undesirable green emission. Unlike previously reported difluorenol molecule, 9,9′-diphenyl-9H,9′H-[2,2′-bifluorene]-9,9′-diol (DPFOH), it was observed that all PL spectra of the isomers, either the solutions or the films, changed conspicuously with the appearance of an additional shoulder of green-band emission under thermal annealing and UV irradiation oxidation (Liu Y. et al., 2020). Compared to DOHSBF, another difluorenol bulky molecule with the similar structure, 2O8-DPFOH-SFX, its raceme and mesomer display the distinct conformations and optoelectronic properties in their condensed states. The annealed *meso*-2O8-DPFOH-SFX film has a stronger green emission band at 510 nm with an I_green_/I_blue_ ratio of 0.5, but no obvious change in the green band was observed for the annealed *rac*-2O8-DPFOH-SFX film (I_green_/I_blue_ = 0.143), indicating stereoisomerism sensitive PL property (Yu et al., 2019). In this regard, only the DOHSBF molecules, either isomers or the mixture, show stereoisomer-insensitive deep blue emission with enhanced stability. Therefore, in spite of the diverse diastereomers, a similar conjugated backbone plays a more key role in dominating optical properties in accordance with the identical absorption and emission spectra of stereoisomers in solution and film states.

**TABLE 1:** Photophysical properties of the isomers at various states.

Entry	*T*_*d*_ (°C)	*T*_*g*_ (°C)	State	*λ*_abs,max_ (nm)	*λ*_PL,max_ (nm)
DOHSBF-mixture	316	114	Solution	333	371, 392,410
			Pristine film	338	423, 443, 471
			Annealed film (200°C)	344	423, 443,471
			Annealed film (220°C)	340	424, 442, 474
			Photooxidation film	339	423, 443, 475
DOHSBF-1	238	-	Solution	333	371, 390, 410
			Pristine film	340	423, 443, 472
			Annealed film (200°C)	344	423, 443, 471
			Annealed film (220°C)	341	424, 444, 475
			Photooxidation film	339	423, 443, 475
DOHSBF-2	229	-	Solution	333	372, 390, 410
			Pristine film	342	423, 443, 472
			Annealed film (200°C)	341	423, 443, 471
			Annealed film (220°C)	345	424, 444, 475
			Photooxidation film	338	423, 443, 475
DOHSBF-3	241	-	Solution	333	371, 390,410
			Pristine film	337	423, 443, 472
			Annealed film (200°C)	338	423, 443, 471
			Annealed film (220°C)	338	424, 444, 475
			Photooxidation film	336	423, 443, 475

For profound understanding of the photophysical properties, then we measured the photoluminescence quantum yield (PLQY) of solutions, pristine films, and annealed films using an integrating sphere. The PLQY values of DOHSBF dilute solutions are relatively high (all four are about 80%) owing to the single-molecular excitonic behavior. Accordingly, the fluorescence PLQY of DOHSBF-mix pristine and annealed films were measured to be Φ_f_ = 13 and 18%, respectively, much lower than that of the solution state, owing to the aggregation-caused quenching (ACQ). The PLQYs of pure stereoisomeric analogues were also measured, with Φ_f_ of 12 and 14% for isomer-1 pristine and the annealed film, 13 and 16% for isomer-2 pristine and the annealed film, 14 and 20% for isomer-3 pristine and the annealed film, respectively. Compared to the initial films, the annealed one shows higher Φ_f_ values, which may be attributed to molecular geometry optimization and ordering under thermal activation, and thus preventing intermolecular exciton coupling and excimer emission. Accordingly, the annealing enhanced fluorescence quantum yield also contributes to stable fluorescence emission.

## Conclusion

In summary, we demonstrated the stereoisomeric effects of DOHSBF on the photophysical behavior. Impressively, the stereoisomeric effect on optical properties in solutions, spin-coated films, and post-treated film states is negligible. As compared to the difluorenols reported in previous literature, we found that DOHSBF in each form exhibit excellent blue spectral stability without undesirable green emission. Spatial isomerism in organic molecule of stereoisomers can not only precisely uncover the structure–function relationship but also play a key role in opening up new design strategies for organic functional materials.

## Data Availability

The original contributions presented in the study are included in the article/Supplementary Material; further inquiries can be directed to the corresponding authors.
