# Supplementary figures and images for: Stereoisomer-Independent Stable Blue Emission in Axial Chiral Difluorenol
Source: Front Chem. 2021 Sep 3;9:717892. doi: 10.3389/fchem.2021.717892 (PMC8446198; doi:10.3389/fchem.2021.717892)

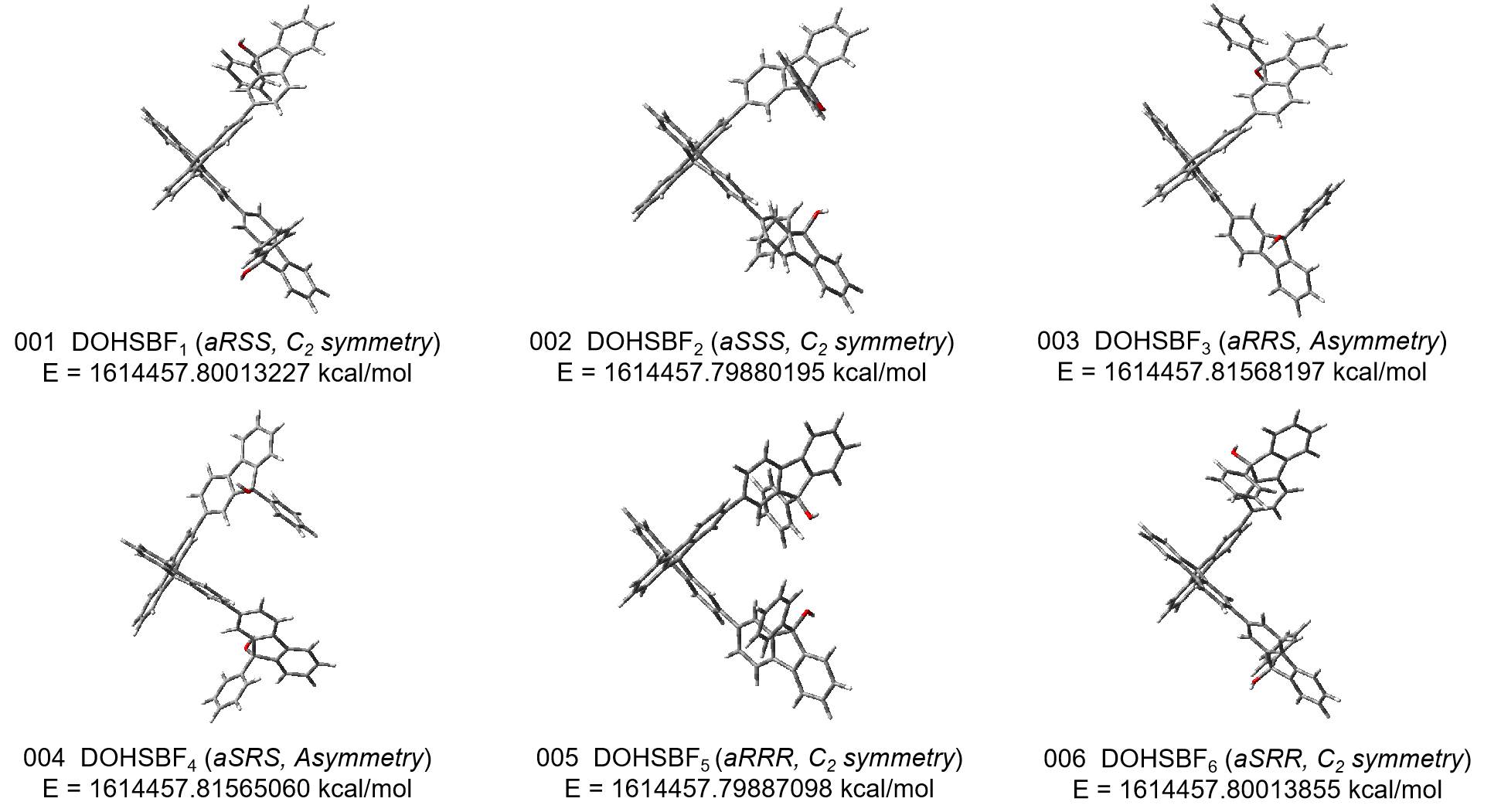

Supplement: Supplementary file 1 [file Image1.tif]
