# Supplementary material for: Stereoisomer-Independent Stable Blue Emission in Axial Chiral Difluorenol
Source: Front Chem. 2021 Sep 3;9:717892. doi: 10.3389/fchem.2021.717892 (PMC8446198; doi:10.3389/fchem.2021.717892)
Supplement: Supplementary file 2 [file DataSheet1.pdf]

Supplementary Material

1 General information

All the solvents and reagents were purchased from commercial suppliers and used without further purification, unless noted otherwise. Spectrochemical-grade solvents were used for optical measurements. 2,2'-dibromo-9,9'-spirobi[fluorine] were obtained from Aldrich Chemical Co. 2-(4,4,5,5-tetramethyl-1,3,2-dioxaborolan-2-yl)-9-(4-(octyloxy)phenyl)-fluoren-9-ol were obtained according to the reported literature. See: (1) Wang L, Zhang G W, Ou C J, Xie L H , Lin J Yi, Liu Y Y, Huang W. Org. Lett. 2014, 16, 1748-1751.

2 Characterization

^{1}H NMR and ^{13}C NMR were recorded on a Bruker 400 MHz and 100 MHz spectrometer in $CDCl_3$ with tetramethylsilane (TMS) as the interval standard. Mass spectra were recorded on a Shimadzu GC-MS 2010 PLUS. For the MALDI-ToF-MS spectra, the spectra were recorded in reflective mode, and substrates were used. The absorption spectra of the solutions and pristine films before and after annealing were measured using a Shimadzu UV-3600 spectrometer (Shimadzu Corporation, Nanjing, Jiangsu, China) at room temperature, and the emission spectra were recorded using a Shimadzu RF-530XPC luminescence spectrometer (Shimadzu Corporation, Nanjing, Jiangsu, China) after excitation at 340 nm. The quartz cells of 10 mm thickness were used to measure the spectra of the dilute solutions. The thin films were prepared by spin-coating on a quartz plate from the tetrahydrofuran solution (concentration: 5 mg/mL) using KW-4A (from the Institute of Micro-Electronics of Chinese Academy of Science) at a rate of 1500 rpm for 60 s. The annealing films were prepared by spin-coated from tetrahydrofuran solutions (concentration: 5 mg/mL) onto the quartz plates and then thermally annealed in air at 220 °C for 10 min.

3 General procedures for synthesis of DOHSBF-mix

Synthesis of 2,2'-(9,9'-spirobi[fluorene]-2,2'-diyl)bis(9-(4-(octyloxy)phenyl)-9H-fluoren-9-ol) (DOHSBF-mix) :

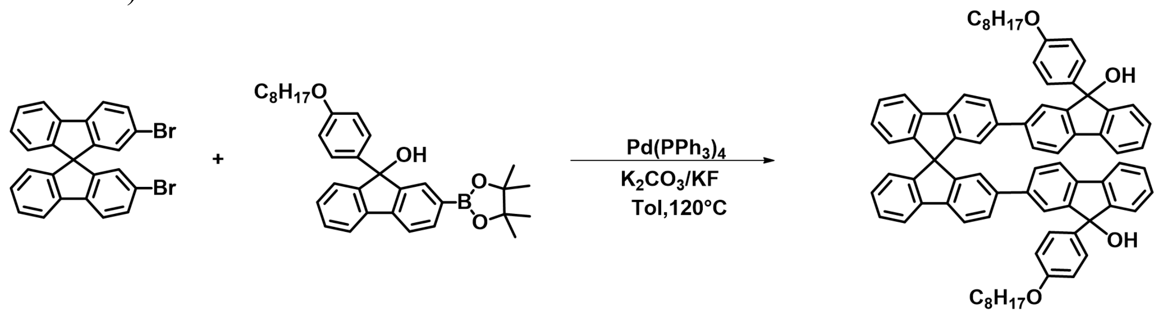

In a two-necked schlenk flask (250 mL), 2,2'-dibromo-9,9'-spirobi[fluorene] (1.5 g, 3.18 mmol), 3 (4.86 g, 9.54 mmol), $Pd(PPh_3)_4$ (220 mg, 228 mmol) were added. The flask was evacuated and back-filled with nitrogen atmosphere over three times, after which degassed toluene (120 mL) and K_2CO_3 / KF aqueous solution (2 M, 30 mL, 2 eq) were injected into the flask through syringe. The mixture was heated up to 120 °C and stirred for 12 hours. The mixture was extracted three times with CH_2Cl_2 and the organic layer was dried over Na_2SO_4, filtered and the solvent was removed under reduced

pressure. Purification by silica gel column chromatography (petroleum ether: CH_2Cl_2 = 1:1.5) to afford DOHSBF-mix (2.66 g, 77%) as a white powder. ^{1}H NMR (400 MHz, $CDCl_3$) δ 7.92-7.85 (m, 4H), 7.66-7.56 (m, 6H), 7.49-7.37 (m, 6H), 7.36-7.29 (m, 4H),7.27-7.19 (m, 6H), 7.15-7.07 (m, 2H), 7.03-6.95 (m, 2H), 6.78-6.70 (m, 6H), 3.90-3.84 (m, 4H), 2.46-2.37 (m, 2H), 1.75-1.65 (m, 4H), 1.43-1.38 (m, 4H), 1.34-1.30 (m, 16H), 0.94-0.82 (m, 6H). ^{13}C NMR (100 MHz, $CDCl_3$) δ 158.4, 151.1, 150.9, 149.2, 141.3, 140.7, 138.9, 138.6, 134.8, 129.0, 128.3, 128.2, 127.8, 127.1, 126.5, 124.6, 124.1, 123.3, 122.6, 120.4, 120.2, 120.1, 114.2, 83.4, 67.9, 66.1, 31.8, 29.4, 29.2, 26.1, 22.7, 14.1. MALDI-ToF-MS: m/z calcd for $C_{79}H_{72}O_4$:1084.56; found: 1084.68.

DOHSBF-1

^{1}H NMR (400 MHz, $CDCl_3$) δ 7.90-7.85 (m, 4H), 7.60-7.58 (d, *J* =8.1 Hz, 4H), 7.57-7.55(d, *J* =7.8 Hz, 2H), 7.46- 7.36 (m, 6H), 7.34-7.26 (m, 4H), 7.25-7.16 (m, 6H), 7.12-7.08 (t, *J* = 6.5 Hz, 2H), 6.96 (s, 2H), 6.76-6.71 (m, 6H), 3.87-3.84 (t, *J* = 6.4 Hz, 4H), 2.39 (s, 2H), 1.75-1.65 (m, 4H), 1.43-1.38 (m, 4H), 1.34-1.30 (m, 16H), 0.94-0.82 (m, 6H).^{13}C NMR (100 MHz, $CDCl_3$) δ 158.4, 151.0, 150.9, 149.2, 141.3, 140.6, 138.9, 138.6, 134.8, 129.0, 128.3, 128.2, 127.8, 127.1, 126.5, 124.6, 124.1, 123.2, 122.6, 120.3, 120.2, 120.1, 114.2, 83.4, 67.9, 66.1, 31.8, 29.4, 29.2, 26.0, 22.7, 14.1. MALDI-ToF-MS: m/z calcd for $C_{79}H_{72}O_4$: 1084.56; found: 1084.68.

DOHSBF-2

^{1}H NMR (400 MHz, $CDCl_3$) δ 7.91-7.83 (m, 4H), 7.65-7.56 (m, 6H), 7.47-7.36 (m, 6H), 7.34-7.27 (m, 4H), 7.26-7.17 (m, 6H), 7.13-7.03 (m, 2H), 6.99-6.92 (m, 2H), 6.78-6.72 (m, 6H), 3.89- 3.83 (m, 4H), 2.39 (s, 2H), 1.75-1.65 (m, 4H), 1.43-1.38 (m, 4H), 1.34 -1.30 (m, 16H), 0.94-0.82 (m, 6H). ^{13}C NMR (100 MHz, $CDCl_3$) δ158.4, 151.1, 150.9, 149.2, 141.3, 140.7 , 138.9, 138.7 , 134.8, 129.0, 128.3, 127.8, 127.1, 126.6, 124.6, 124.1, 123.3, 122.7, 120.3, 120.2, 120.1, 114.2, 83.4, 67.9, 66.1, 31.8,29.4, 29.2, 26.1, 22.7, 14.2. MALDI-ToF-MS: m/z calcd for $C_{79}H_{72}O_4$: 1084.56; found: 1084.55.

DOHSBF-3

^{1}H NMR (400 MHz, $CDCl_3$) δ 7.93-7.85 (m,4H), 7.66-7.57 (m, 6H), 7.46-7.40 (m, 4H), 7.38-7.27 (m, 6H), 7.27-7.20 (m, 6H), 7.12-7.09 (t, *J* = 7.6 Hz, 2H), 7.00 (s, 2H), 6.80- 6.70 (m, 6H), 3.88-3.85 (t, *J* = 6.5 Hz, 4H), 2.44 (s, 2H), 1.75-1.65 (m, 4H), 1.43-1.38 (m, 4H), 1.34-1.30 (m, 16H), 0.94-0.82 (m, 6H). ^{13}C NMR (100 MHz, $CDCl_3$) δ 158.4, 151.1, 150.9, 149.2, 141.3, 140.7, 139.0, 138.6, 134.8, 129.0, 128.3, 128.2, 127.9, 127.8, 127.1, 126.5, 124.6, 124.1, 123.3, 122.7, 120.4, 120.1, 114.1, 83.4, 67.9, 66.1, 31.8, 29.4, 29.2,26.0, 22.7, 14.1. MALDI-ToF-MS: m/z calcd for $C_{79}H_{72}O_4$: 1084.56; found: 1084.78.

4 Theoretical energy calculation data

Energy calculation comparison of possible theoretical stereoisomers of DOHSBF (alkyl chains are omitted) by Gaussian 09 at the B3LYP/6-31G(d) level.

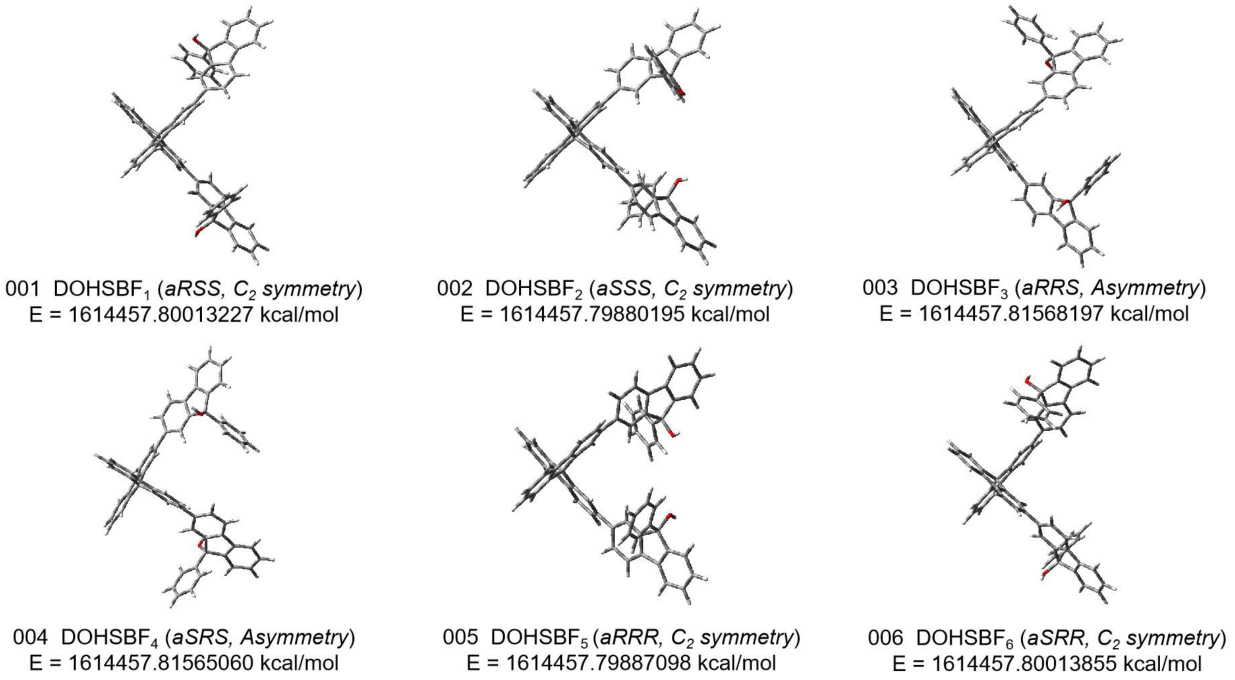

8

Supplementary Figure 1. Energy calculation of possible theoretical stereoisomers of DOHSBF (alkyl chains are omitted).

5 Supplementary Figures

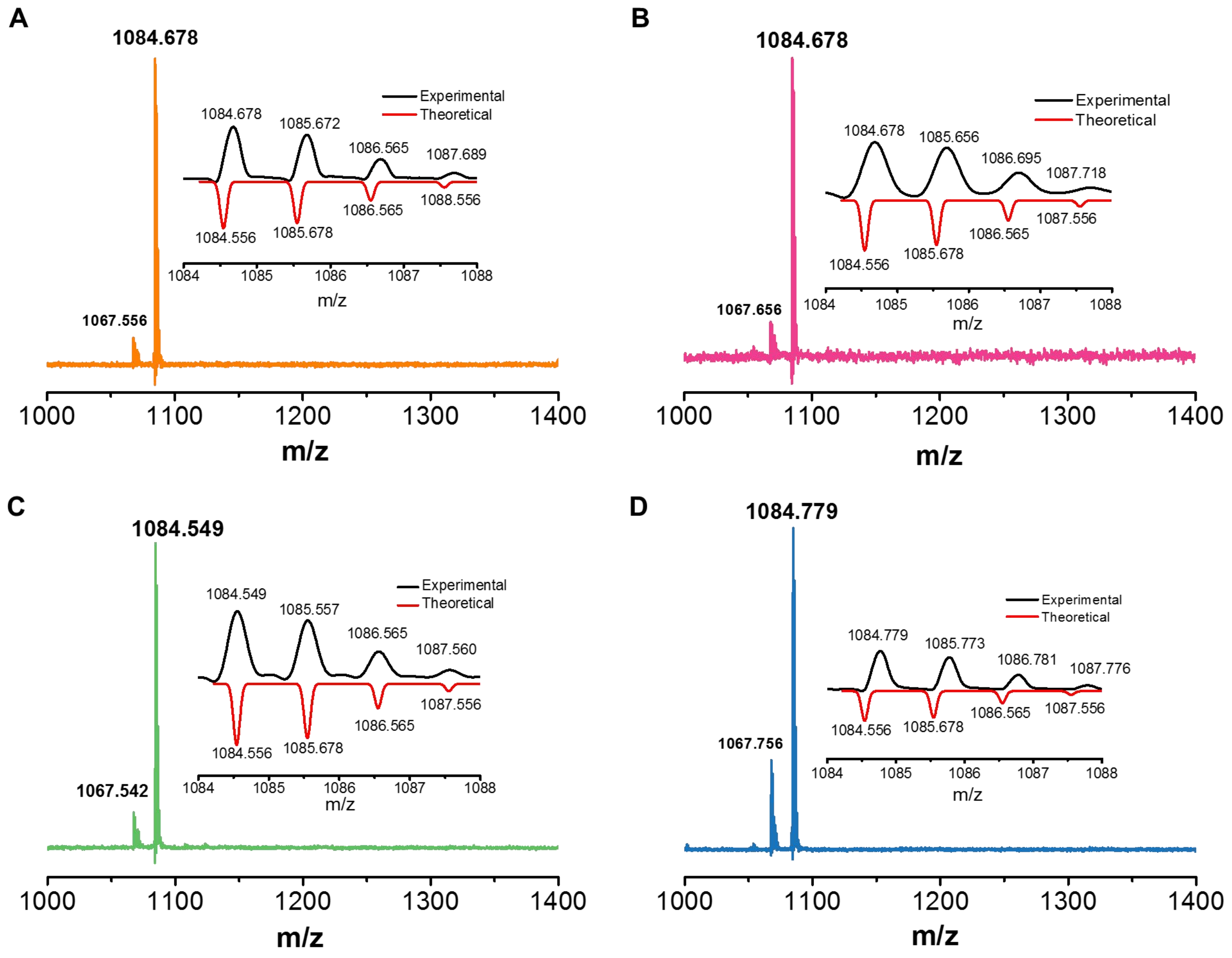

Supplementary Figure 2. MALDI-ToF-MS spectra of (A) DOHSBF-mix, (B) DOHSBF-1, (C) DOHSBF-2 and (D) DOHSBF-3, respectively.

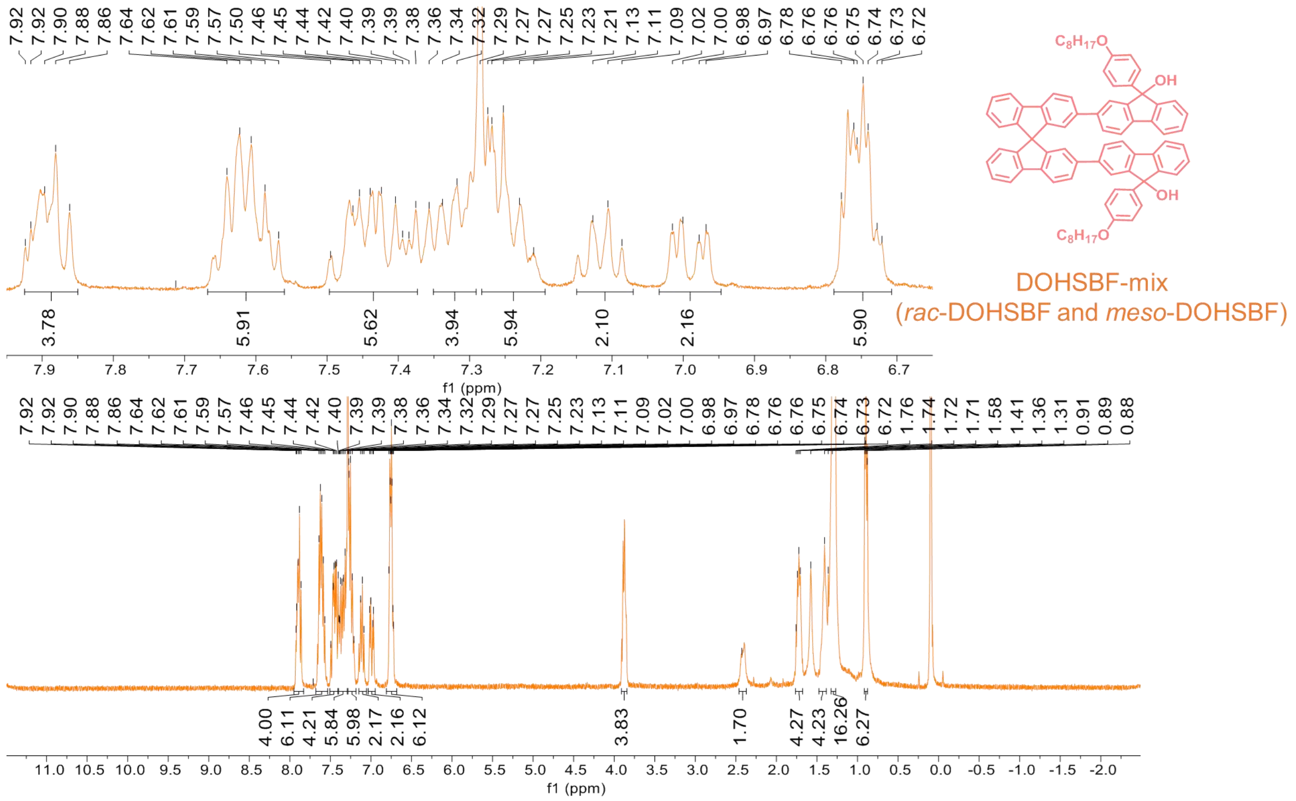

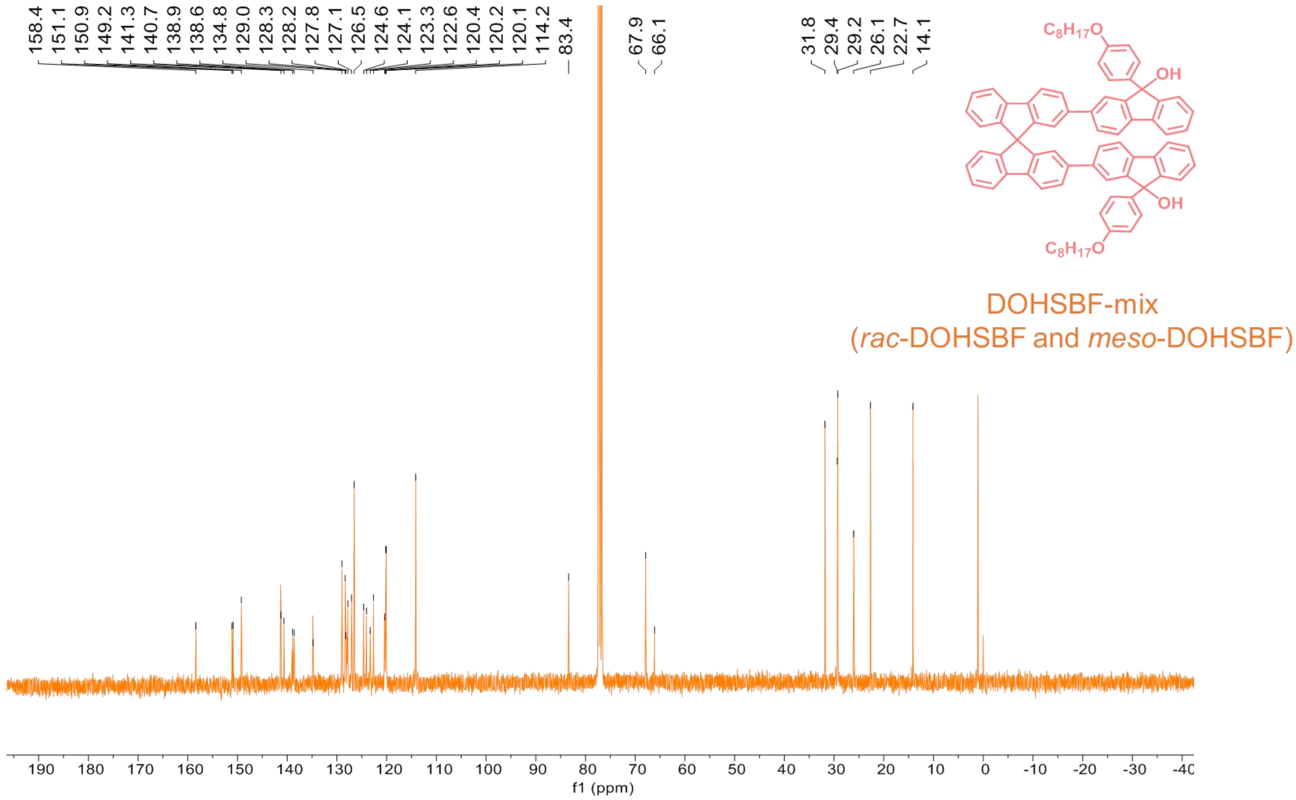

Supplementary Figure 3. ^{1}H NMR and ^{13}C NMR spectra of DOHSBF-mix in $CDCl_3$.

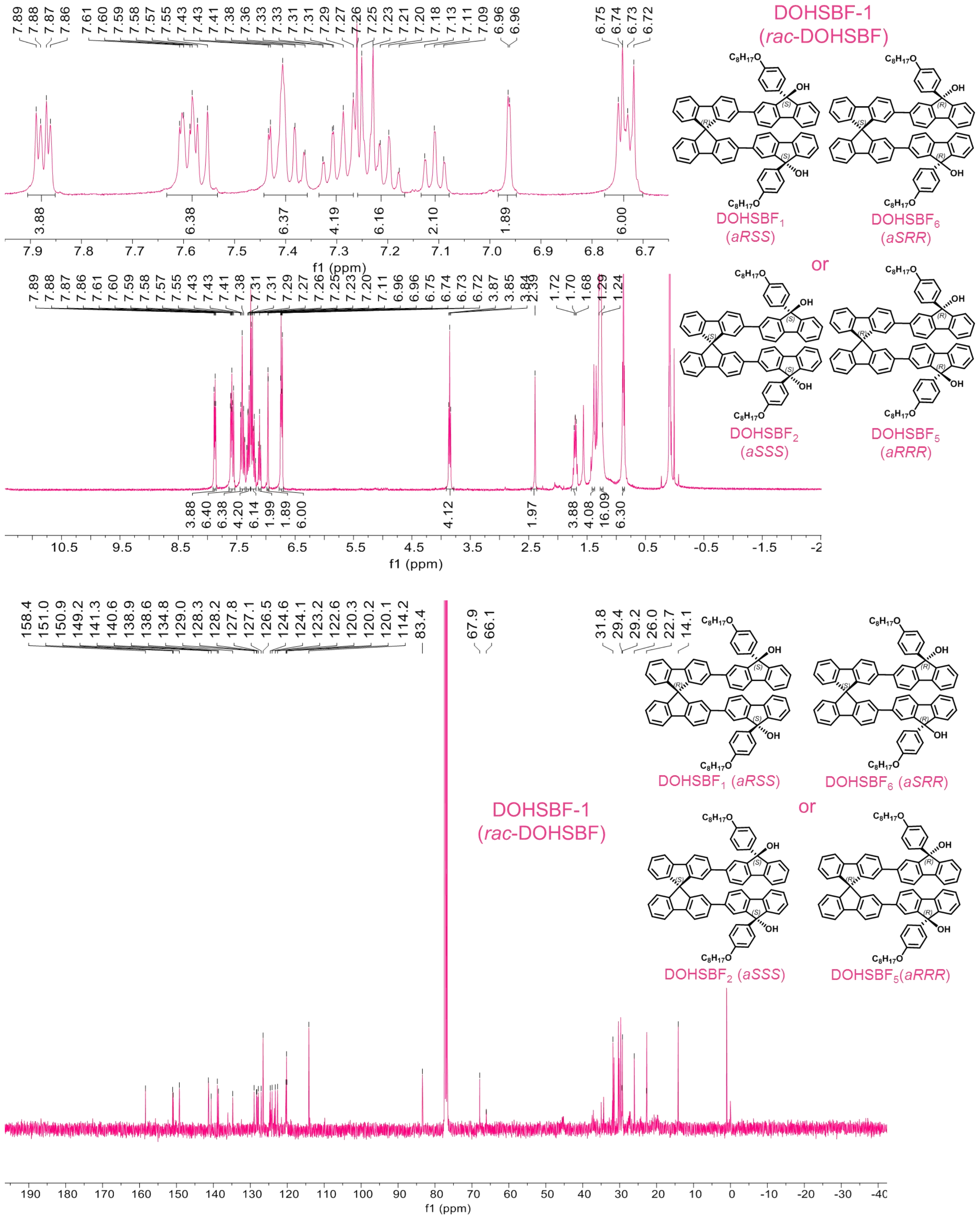

Supplementary Figure 4. ^{1}H and ^{13}C NMR spectra of DOHSBF-1 in $CDCl_3$.

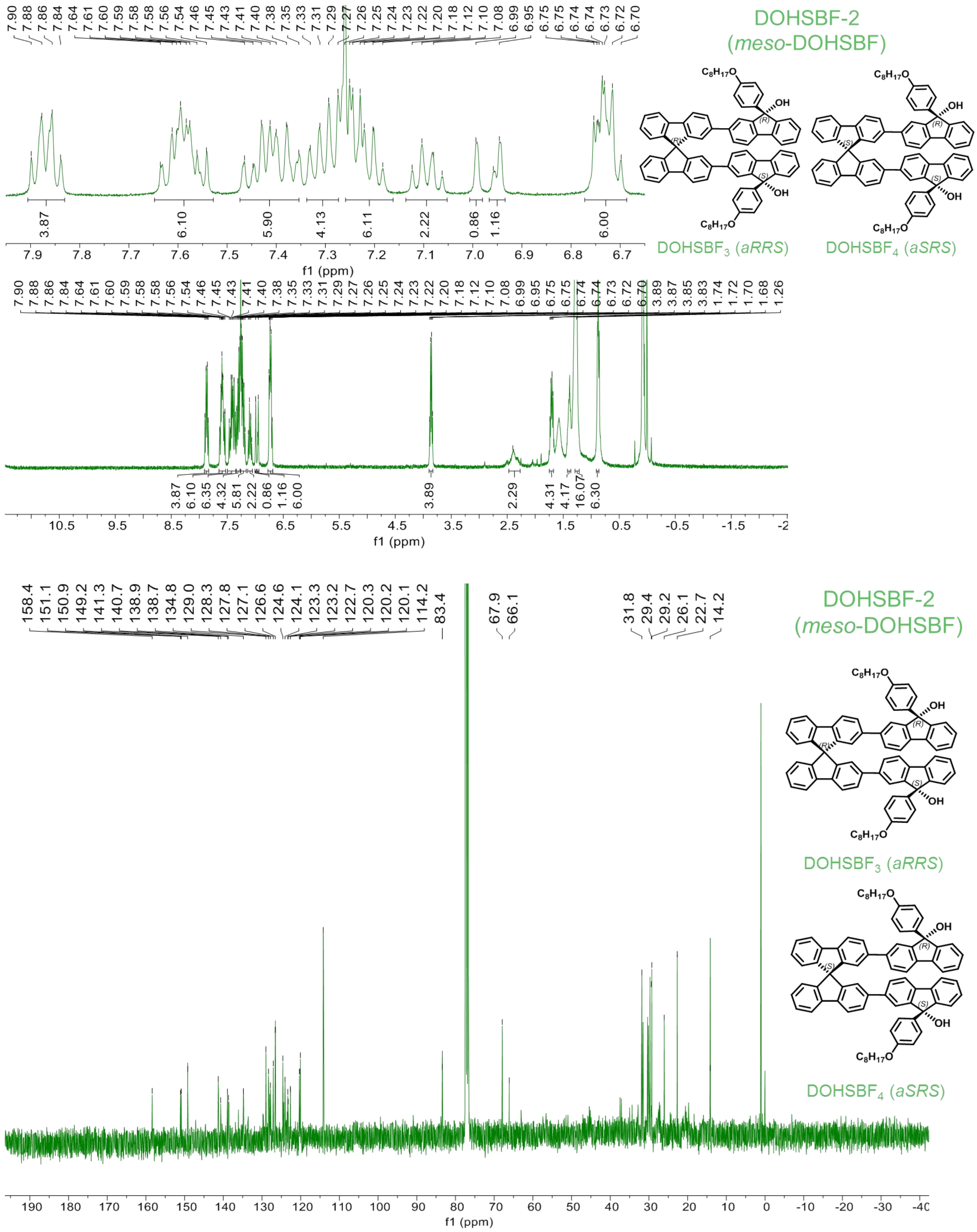

Supplementary Figure 5. ^{1}H and ^{13}C NMR spectra of DOHSBF-2 in $CDCl_3$.

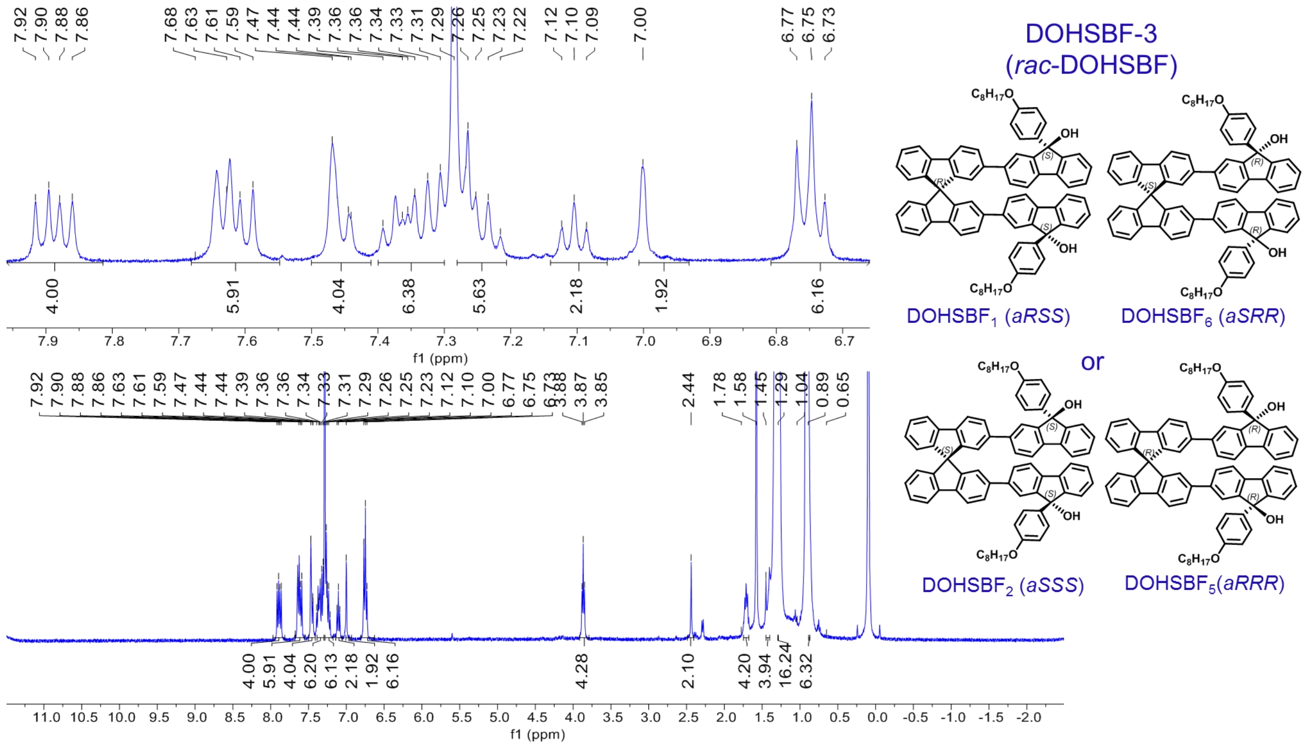

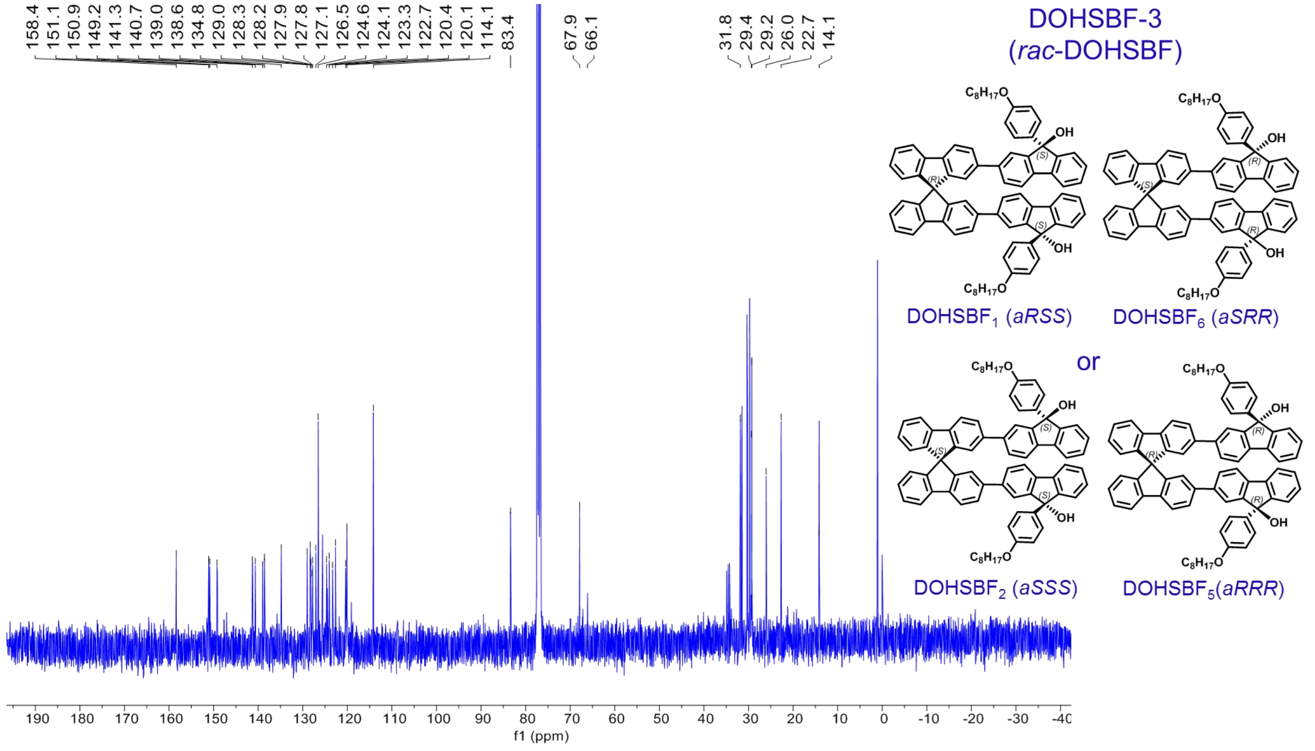

Supplementary Figure 6. ^{1}H and ^{13}C NMR spectra of DOHSBF-3 in $CDCl_3$.

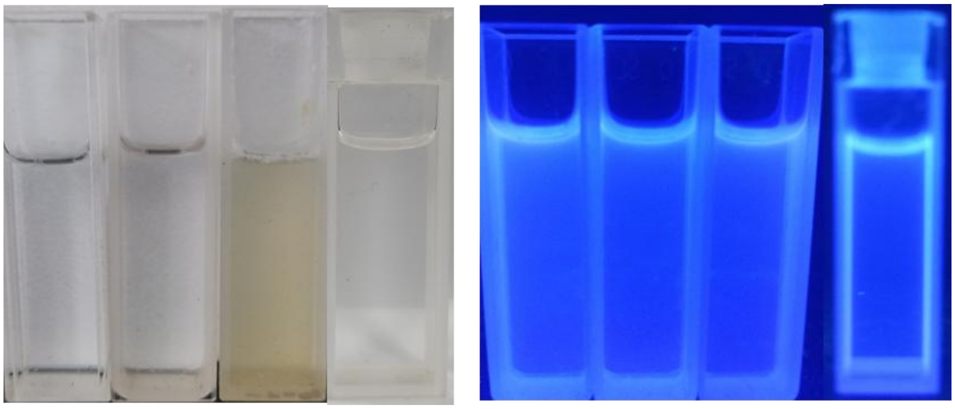

Supplementary Figure 7. The solubility of DOHSBF-1, DOHSBF-2, DOHSBF-3 and DOHSBF-mix in dichloromethane and the corresponding photographs of solutions with different concentrations under sunlight (left), 365 nm UV light (right).

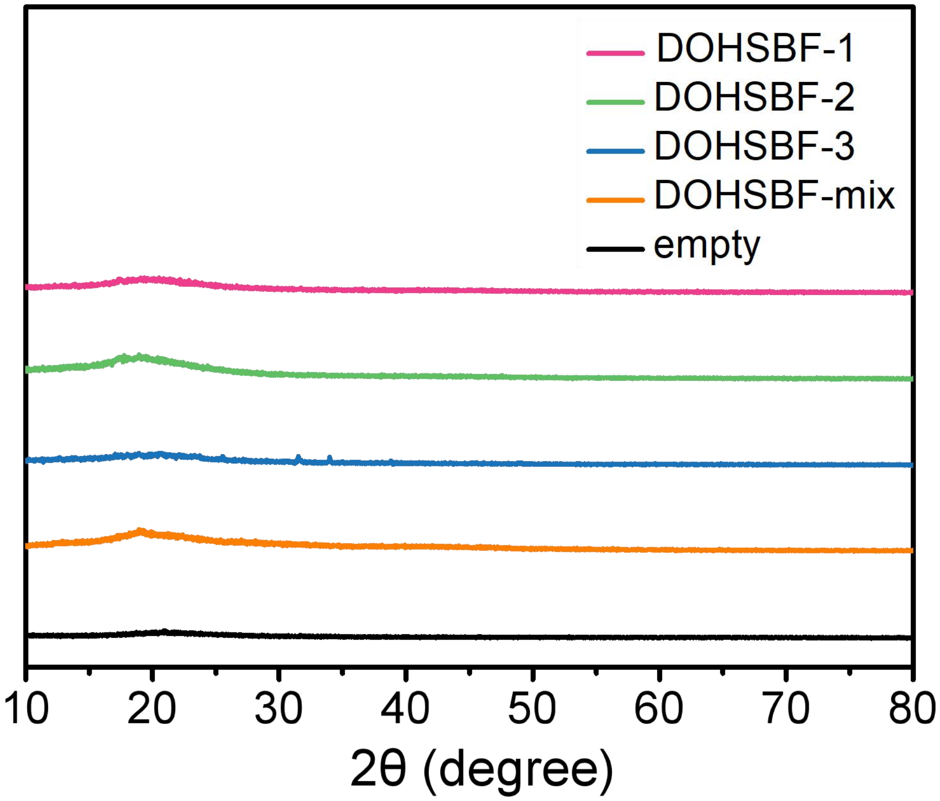

Supplementary Figure 8. XRD curves of DOHSBF in powder state.

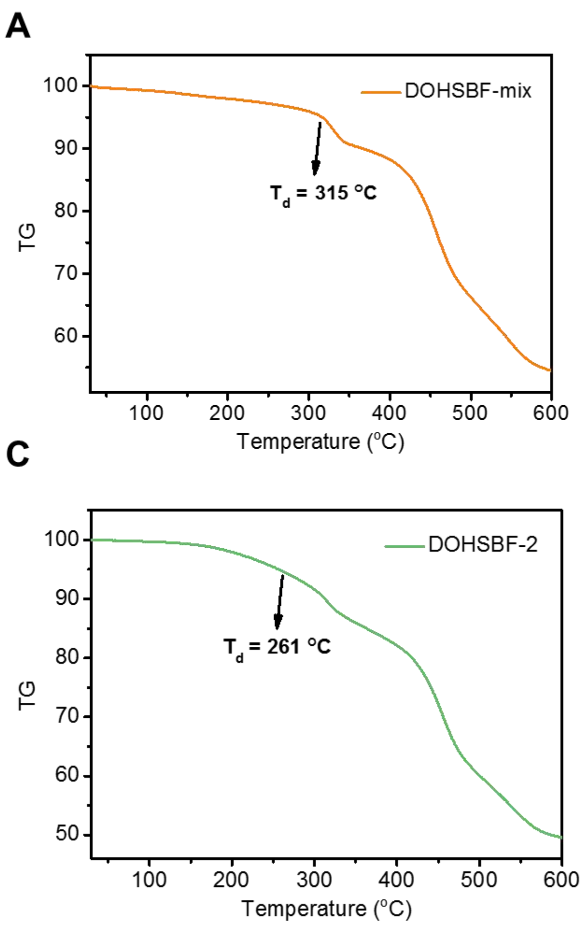

Supplementary Figure 9. TG curves of DOHSBF in each form.

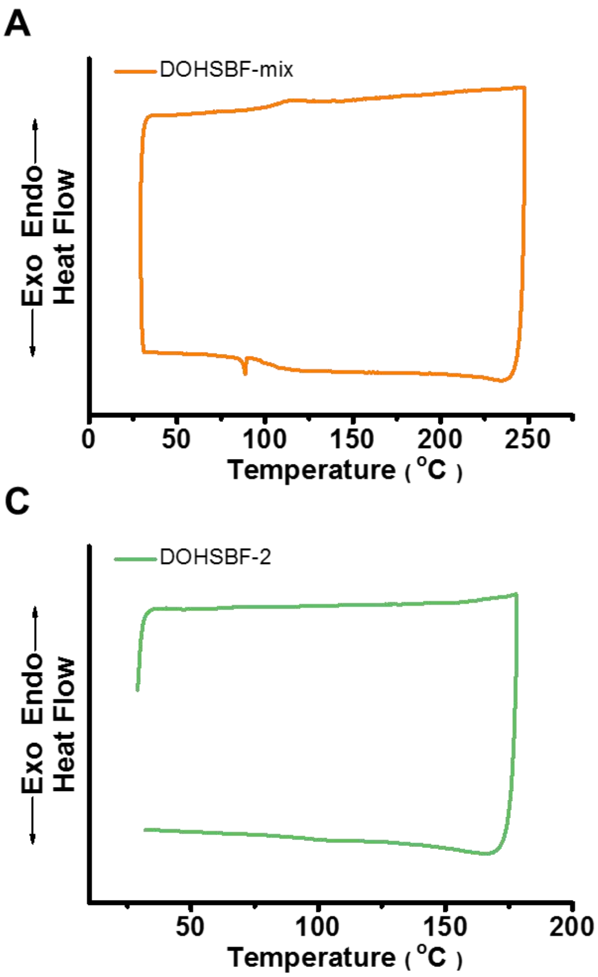

Supplementary Figure 10. DSC curves of DOHSBF in each form.

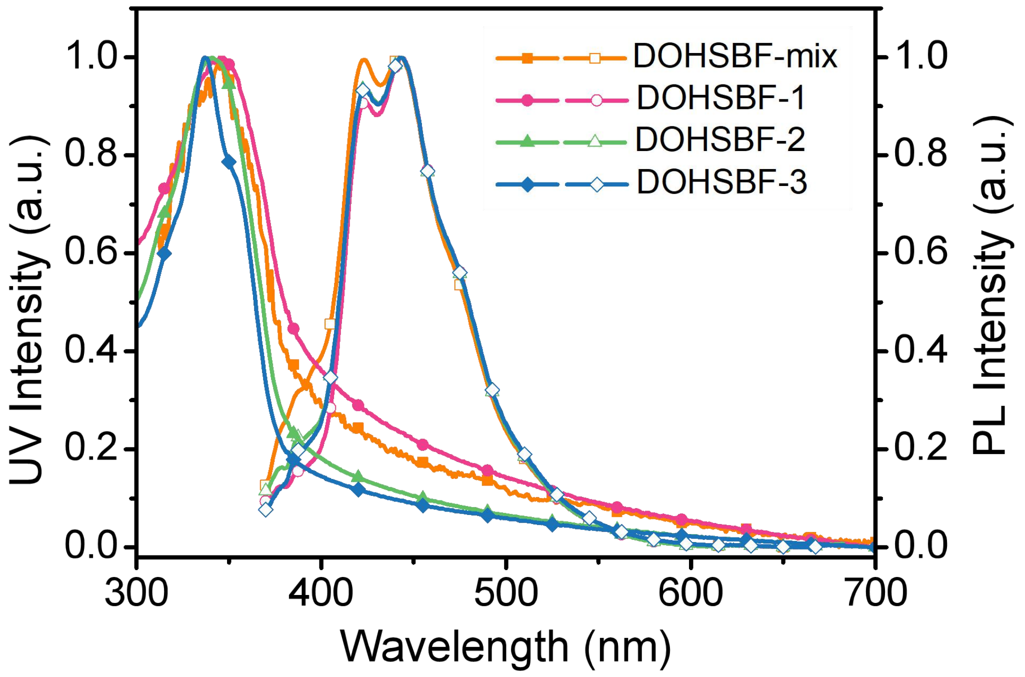

Supplementary Figure 11. UV-vis absorption and PL spectra of DOHSBF annealed film at 200 °C for 10 min.

Table S1. Electrochemical properties of DOHSBF in different forms.

	Oxidation onset potential (V)	Reduction onset potential (V)	E_{HOMO} (eV)	E_{LUMO} (eV)	E_g (eV)
DOHSBF-mix	1.49	-2.02	-6.26	-2.75	3.51
DOHSBF-1	1.54	-2.07	-6.31	-2.70	3.61
DOHSBF-2	1.50	-2.14	-6.27	-2.63	3.64
DOHSBF-3	1.48	-2.10	-6.25	-2.67	3.58
